# Correction: Recovery and partial isolation of α-mangostin from mangosteen pericarpsvia sequential extraction and precipitation

**DOI:** 10.1371/journal.pone.0342249

**Published:** 2026-02-03

**Authors:** Moh Moh Han, Preuk Tangpromphan, Amaraporn Kaewchada, Attasak Jaree

The Acknowledgement section for this article is missing. The Acknowledgement section is: This project was funded by the Faculty of Engineering, Kasetsart University, Thailand (grant ID: 65/04/CHEM).

In the Funding statement, the grant ID from the funder Faculty of Engineering, Kasetsart University, Thailand is missing. The grant ID is: 65/04/CHEM.
