# Supplementary material for: Recovery and partial isolation of ⍺-mangostin from mangosteen pericarpsvia sequential extraction and precipitation
Source: PLoS One. 2024 Oct 25;19(10):e0310453. doi: 10.1371/journal.pone.0310453 (PMC11508469; doi:10.1371/journal.pone.0310453)
Supplement: S1 File — (DOCX) [file pone.0310453.s001.docx]

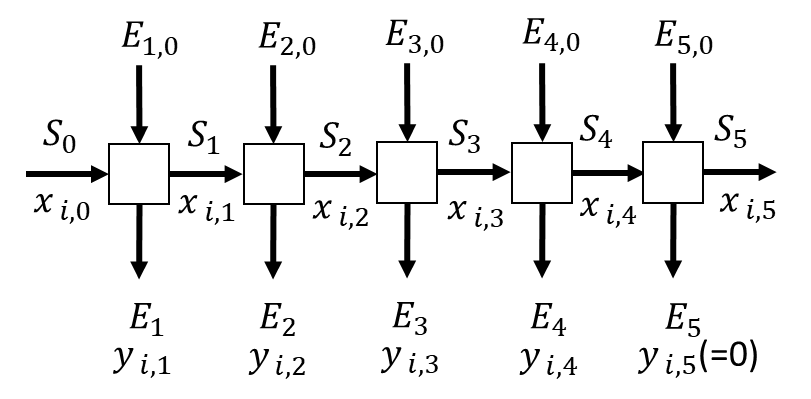


**Fig 1.** Crosscurrent extraction


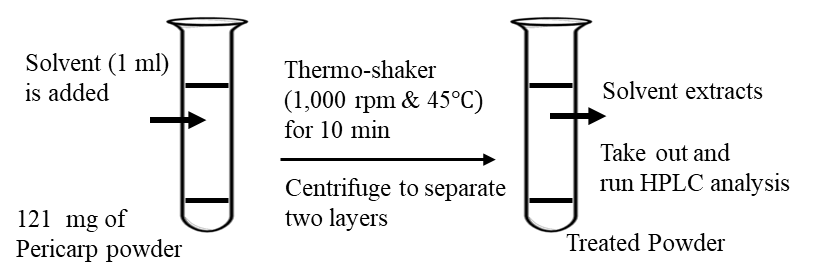

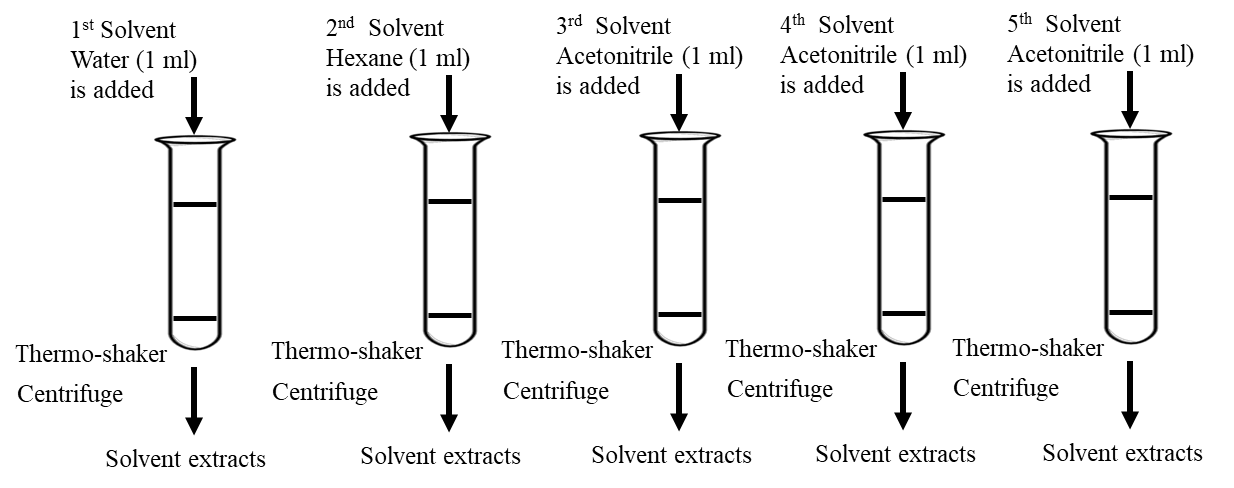


**Fig 2.** Sequential extraction method


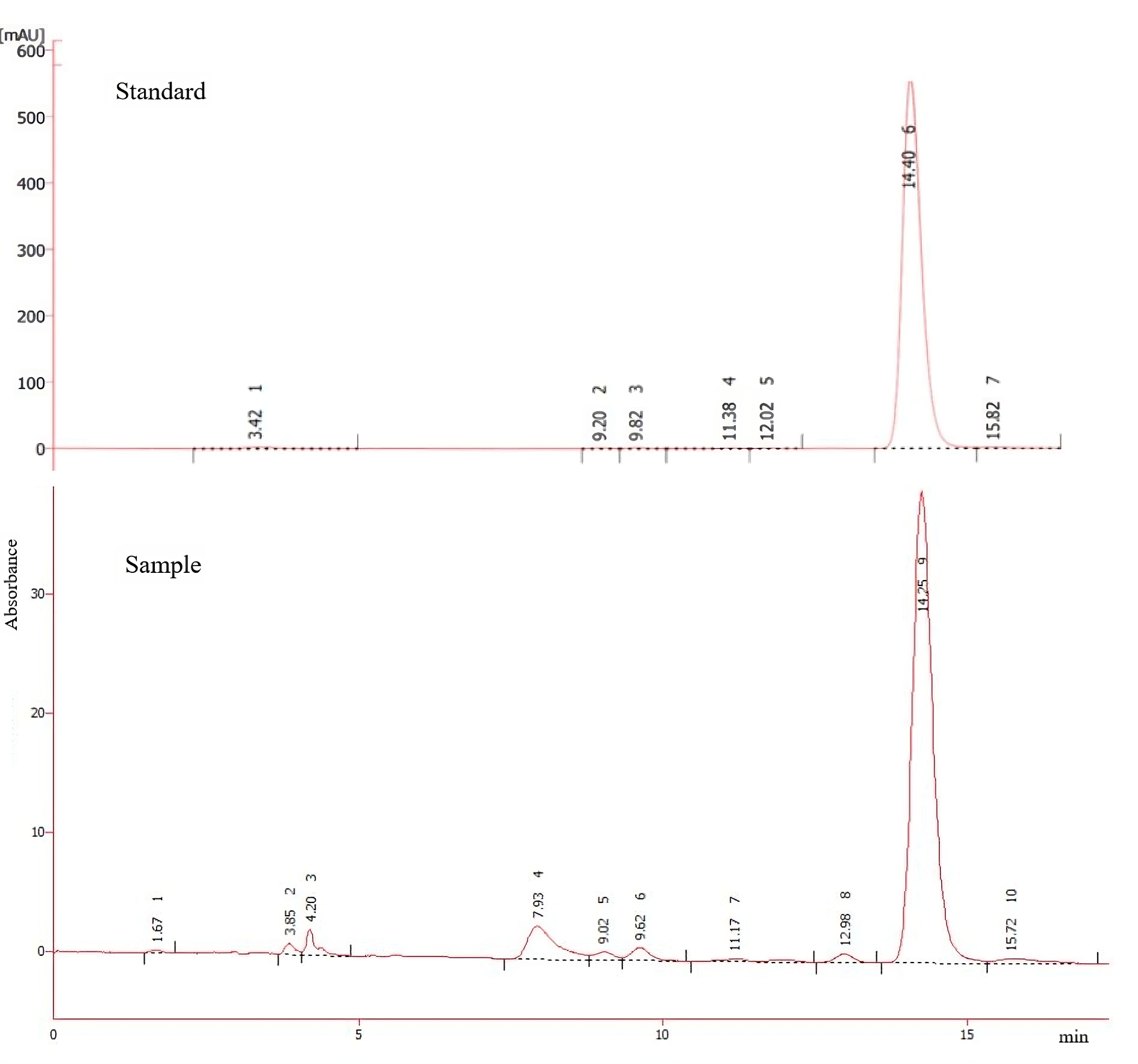


(b)

(a)

**Fig 3.** Chromatogram of (a) standard of ⍺-M (b) extract solution obtained from 1^st^-step percolation using acetonitrile as solvent.

*
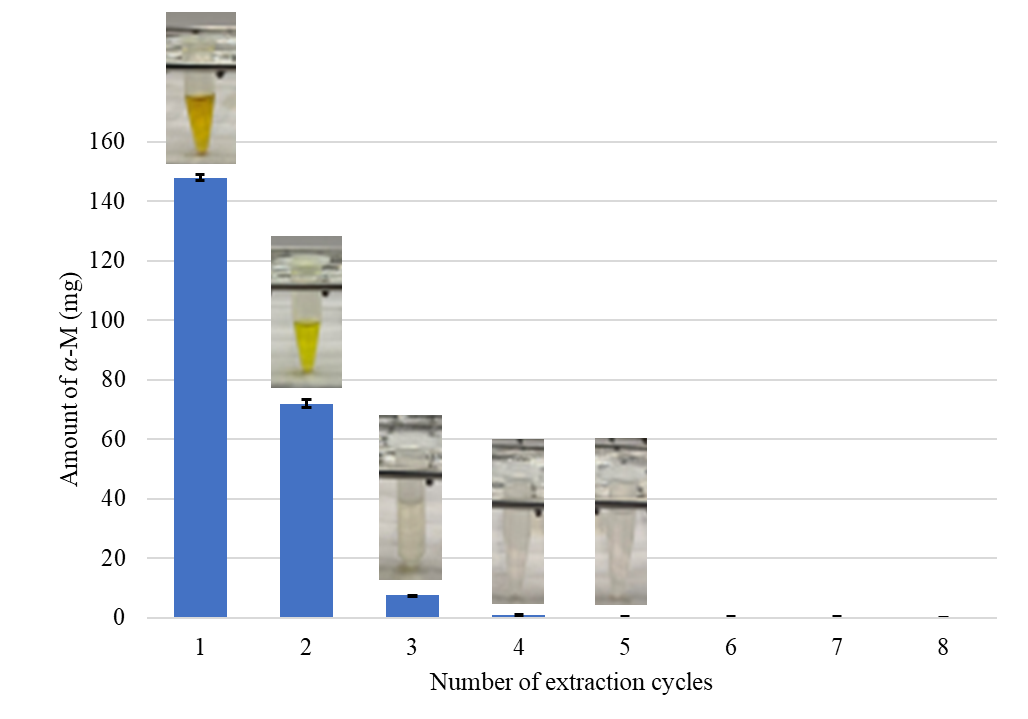
*

**Fig 4.** Amounts of ⍺-M obtained from multi-stage percolation.

| **No** | **Amount of alpha 1** | **Amount of alpha 2** | **Average of Amount of Alpha** | **Std. Dev** |
| --- | --- | --- | --- | --- |
| 1 | 148.553 | 147.2640 | 147.9086 | 0.645 |
| 2 | 70.979 | 72.9021 | 71.9404 | 0.962 |
| 3 | 7.522 | 7.3774 | 7.4499 | 0.072 |
| 4 | 0.921 | 0.9611 | 0.9410 | 0.020 |
| 5 | 0.281 | 0.3718 | 0.3264 | 0.045 |
| 6 | 0.203 | 0.3226 | 0.2627 | 0.060 |
| 7 | 0.203 | 0.1617 | 0.1822 | 0.021 |
| 8 | 0.062 | 0.0568 | 0.0594 | 0.003 |


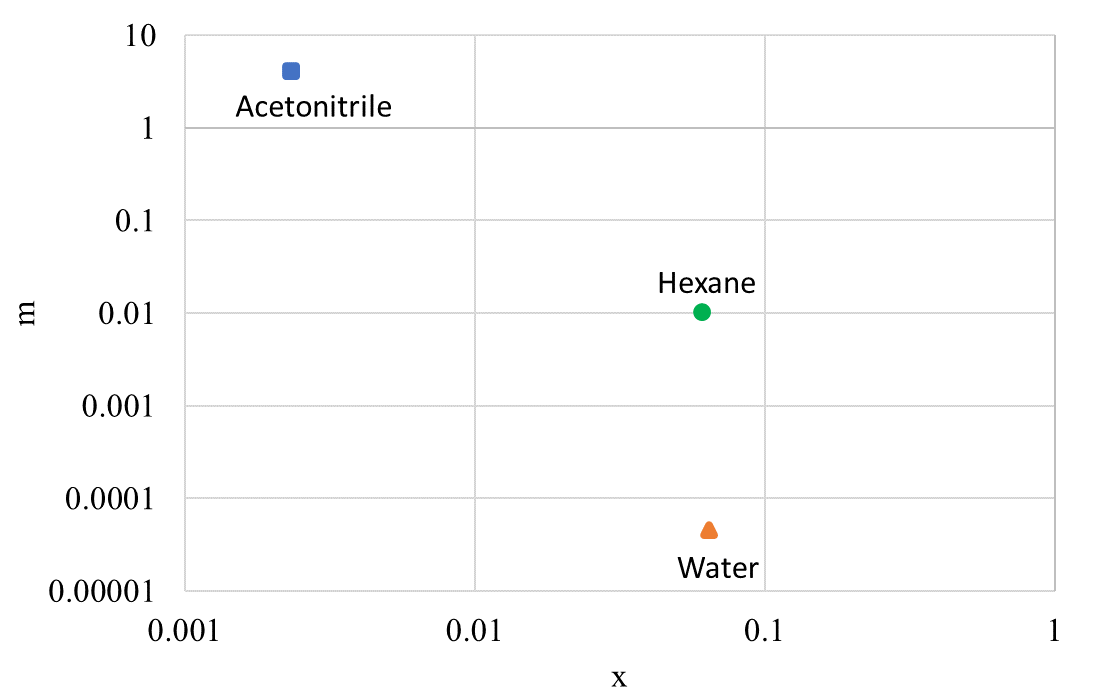


**Fig 5.** Solvent distribution factor of acetonitrile, hexane, and water.

| **Solvents** | **xi** | **mi** |
| --- | --- | --- |
| Acetonitrile | 0.002331 | 4.085219 |
| Hexane | 0.060771 | 0.010361 |
| Water | 0.064176 | 0.000045 |


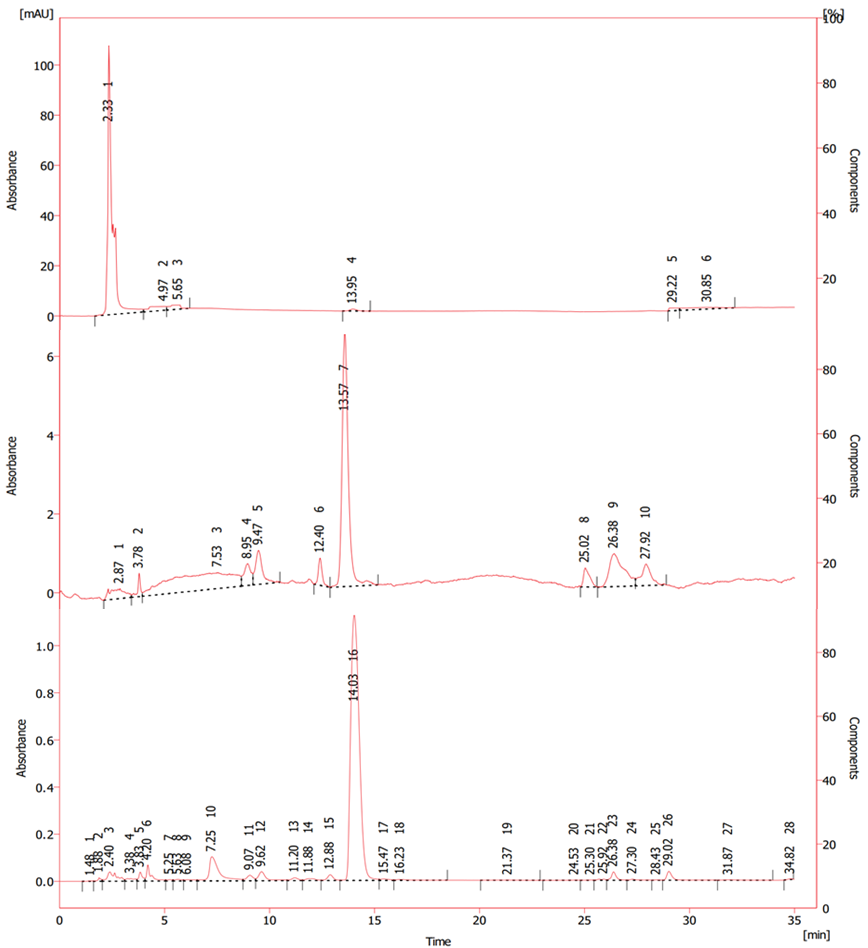


(c)

(b)

(a)

**Fig 6.** Chromatogram of extract solution obtained from different stages of sequential extraction (a) water (b) water-hexane (c) water-hexane-acetonitrile.


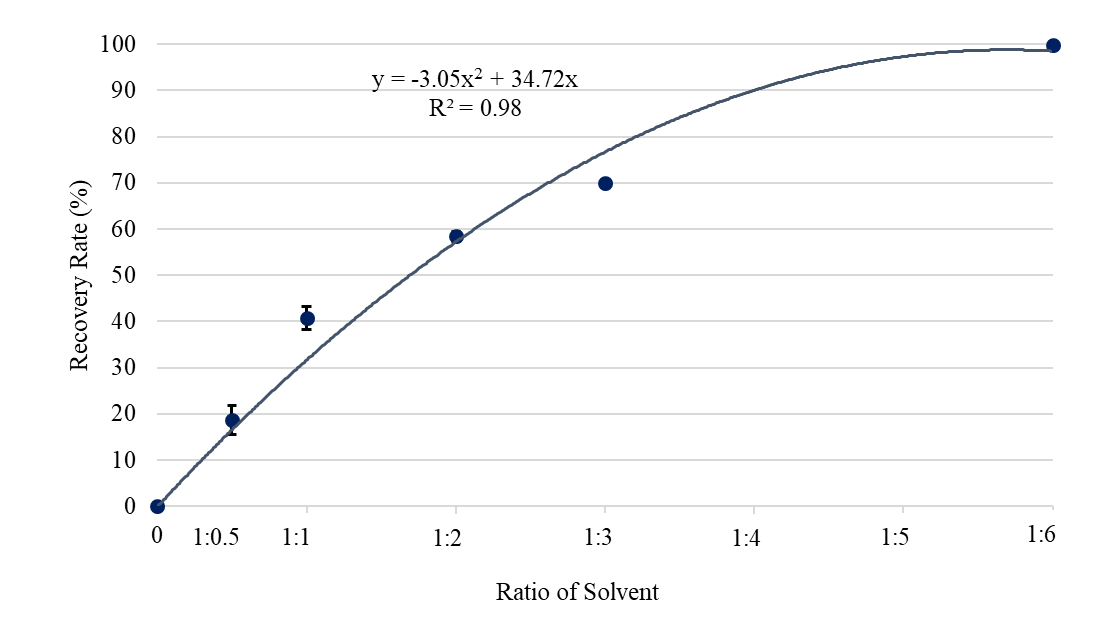


**Fig 7.** Recovery of crude extract via precipitation.

| Ratio of the solvent | Ratio of the solvent | %recovery (1) | %recovery (2) | Average % recovery | Std Dev. |
| --- | --- | --- | --- | --- | --- |
| 1:0 | 0 | 0.00 | 0.00 | 0.00 | 0.00 |
| 1:0.5 | 0.5 | 15.47 | 21.70 | 18.65 | 3.11 |
| 1:1 | 1 | 38.11 | 43.18 | 40.70 | 2.54 |
| 1:2 | 2 | 57.26 | 59.54 | 58.42 | 1.14 |
| 1:3 | 3 | 69.79 | 70.18 | 69.99 | 0.19 |
| 1:6 | 6 | 99.76 | 99.76 | 99.76 | 0.00 |
