# Supplementary material for: Recovery and partial isolation of ⍺-mangostin from mangosteen pericarpsvia sequential extraction and precipitation
Source: PLoS One. 2024 Oct 25;19(10):e0310453. doi: 10.1371/journal.pone.0310453 (PMC11508469; doi:10.1371/journal.pone.0310453)
Supplement: Attachment — Submitted filename: Response to reviewer.docx [file pone.0310453.s002.docx]

Reviewer #1: Manuscript describes isolation of alpha-mangostin from the pericarps in Thailand. The manuscript is very well written with good experimental section. Thus, I would suggest this interesting paper for publication in current form in this journal.

**Response:** Thank you very much for your recommendation.

Reviewer #2: Thank you for the opportunity given to make corrections to the article with the title "Recovery and Partial Isolation of ⍺-Mangostin from Mangosteen Pericarps (Thailand) via Sequential Extraction and Precipitation". Several aspects need to be addressed in this article from background to conclusion. The following points need to be addressed in this article:

1. Introduction

- The explanation of mangostin on the first page is too much, so I suggest combining paragraphs two, three, four, and five into a single short paragraph.

**Response:** We thank the reviewer for their suggestion to improve the conciseness of the mangosteen introduction. We have consolidated paragraphs two through five into a single paragraph to enhance the overall flow of the manuscript.

**Original: (Introduction)**

Mangosteen (Garcinia mangostana L.) is a significant commercial fruit in Thailand and various Southeast Asian countries, often referred to as the "queen of fruits" due to the crown-like appearance of its flowers. Notably, India leads the global production of mangosteens, while Thailand ranks second in Southeast Asia after Indonesia. The fruit's composition comprises 83% pericarps, 15% pulp, and 2% seeds by weight [1]. The pericarps, often considered byproducts, possess 82.50% carbohydrates, 6.45% fat, 3.02% protein, 2.17% ash, and 2.17% free sugars. [2]

Although the pericarp of fresh mangosteen initially holds a modest market value of 0.2 USD per kg, its value significantly increases when functional chemicals, particularly xanthones, are extracted from it. Studies suggest that from 1 kg of fresh mangosteen, approximately 2 g of pure xanthones can be obtained, which are valued at 15 times more than the pericarp itself [3]. Consequently, the extraction and purification procedures for xanthones are necessary for various industries, presenting a marketable product with substantial health advantages.

Mangosteen pericarps are rich in health-promoting phenolic compounds, including tannins, flavonoids, anthocyanins, xanthones, and benzophenones [4]. Xanthones, a subgroup of phenolics, are particularly abundant and are known for their diverse health benefits such as antitumor, anticancer, antibacterial, and antioxidant properties [5]. Approximately 200 varieties of xanthones exist in nature, with about 40 types found in mangosteen, belonging to families such as Bonnetiaceae, Clusiaceae, and Podostemaceae [6].

Extracts from mangosteen pericarp and xanthones find extensive applications across the medical, food supplement, oral care, and cosmetic industries. In medicine, pericarp extracts form the basis for various oral health products targeting fungal itch and ulcers. Within the food supplement sector, mangosteen pericarps are utilized in dietary supplements, including antioxidants and anti-aging products, to enhance immunity. The oral care industry employs pericarp extract in mouthwash, toothpaste, dental floss, and breath spray formulations. In the food industry, it contributes to antioxidant snacks, energy-boosting foods, snack bars, rice powder, and healthy beverages [7]. Furthermore, in cosmetics, pure xanthones derived from mangosteen pericarp are incorporated into skincare products designed to address acne and aging, promoting skin health and radiance [8].

Xanthones, polyphenol compounds abundant in mangosteen pericarp, include more than 68 varieties, with Alpha-mangostin (⍺-M) being the predominant substance (69.01%), followed by Gamma-mangostin (γ-M) (17.86%) and minor compounds. Figure 1 illustrates the chemical structures of xanthones and ⍺-M.

Xanthones can be extracted from either fresh or dried mangosteen pericarp. However, due to issues related to high moisture content and the risk of microbial contamination, dried pericarps are commonly preferred for extraction purposes [9]. Increased antioxidant capacity has been attributed to ⍺-M, a hydrophobic polyphenol compound [10, 11]. Therefore, purification is crucial to ensure the quality of target bioactive compounds, preventing undesirable side effects. The choice of solvent during extraction significantly influences compound purity, with various solvents of different polarities employed based on the characteristics of target compounds.

**Revised:** (Introduction)

Mangosteen (Garcinia mangostana L.), often hailed as the "queen of fruits" due to its distinctive floral appearance, is a commercially significant crop in Thailand and Southeast Asia. While India leads global production, Thailand is a major producer in the region [1]. The fruit comprises 83% pericarps, 15% pulp, and 2% seeds [2]. Although the pericarps have limited initial market value, they possess substantial potential due to their rich content of bioactive compounds, particularly xanthones [3]. These compounds exhibit a wide range of health benefits, including antioxidant and anticancer properties, driving demand across medical, food, cosmetic, and oral care industries [4, 5].

- It is best not to include images in the background

**Response:** We appreciate the reviewer's comment regarding the inclusion of images in the background. In response, we have removed Figure 1, which depicted the molecular structures of xanthone and α-mangostin. We believe this modification enhances the manuscript's focus and clarity.

**Original:**

**Figure 1***. Xanthone and ⍺-mangostin*

**Revised:**

The image has been removed.

- There are no citations showing the percentages of α-mangostin and gamma-mangostin. Should be added

**Response:** We thank the reviewer for pointing out the omission of citations for the percentages of α-mangostin and γ-mangostin. The necessary references have been incorporated into the manuscript to support these values.

**Original:** (Introduction)

Xanthones, polyphenol compounds abundant in mangosteen pericarp, include more than 68 varieties, with Alpha-mangostin (⍺-M) being the predominant substance (69.01%), followed by Gamma-mangostin (γ-M) (17.86%) and minor compounds. Figure 1 illustrates the chemical structures of xanthones and ⍺-M.

**Revised:**

Mangosteen pericarps contain over 68 types of xanthones, with 69.01% of alpha-mangostin (⍺-M) and 17.86% of gamma-mangostin (γ-M) being the predominant compounds. The extraction yield of ⍺-M and γ-M was reported as 33.24 mg/g dried mangosteen (DM) and 8.61 mg/g of DM, respectively [6].

*Wittenauer J, Falk S, Schweiggert-Weisz U, Carle R, Characterisation and quantification of xanthones from the aril and pericarp of mangosteens (Garcinia mangostana L.) and a mangosteen containing functional beverage by HPLC-DAD-MS n. 2012 Food Chem. 134 (1), 445–452.*

- The introduction prepared in this text contains many definitions and meanings. This section should be made more concise by directly explaining the essence of the problem being discussed. Definitions and understandings that are excessive make the reader lose focus on the main problem being conveyed. It's best to just describe the main problem clearly and concisely to attract attention and make it easier for readers to understand.

**Response:** We thank the reviewer for their valuable feedback regarding the introduction. We have revised this section to enhance its focus on the core research problem. By eliminating extraneous definitions and explanations, we have streamlined the content and aimed to provide a clearer and more concise overview of the research gap and its significance.

**Original:** #1 (7^th^ paragraph in Introduction)

Solvent extraction, a technique to isolate desired compounds without dissolving others, is categorized into leaching (solid-liquid extraction) and liquid-liquid extraction. Factors affecting solid-liquid extraction include solvent and solid properties such as size and extraction time [12]. Although temperature affects solubility and diffusion rates, it does not influence viscosity; however, high temperatures may damage the target bioactive compounds. Solid-liquid extraction is commonly employed for extracting active substances from mangosteen pericarp.

**Revised:** #1 (2^nd^ paragraph in Introduction)

Bioactive substances from mangosteen pericarp can be extracted by solid-liquid extraction. While both fresh and dried pericarps can be used for extraction, the latter is preferred to mitigate microbial contamination and high moisture content issues [8]. Factors affecting solid-liquid extraction include solvent and solid properties as well as the operating conditions such as extraction time, and temperature [9]. High temperatures can degrade bioactive compounds despite influencing solubility and diffusion rates.

**Original:** #2 (8^th^ paragraph in Introduction)

The quality of the targeted active compounds generated relies on employing an effective extraction method. The presence of a clearly defined chromatographic peak corresponding to the targeted chemical signifies the purity of the extracted substance. Ensuring the purity of a molecule derived from a plant extract is crucial for preventing undesirable side effects in pharmaceutical or medical uses, as variations in the compound's chemical properties can lead to adverse outcomes.

**Revised:** #2 (3^rd^ paragraph in Introduction)

The quality of extracted bioactive compounds depends on an effective extraction method, with a clear chromatographic peak indicating purity. Ensuring purity is crucial to prevent side effects in pharmaceutical or medical applications, as chemical variations can lead to adverse outcomes.

- Currently, the paragraph describes extraction, purification, and then back to extraction. This order needs to be rearranged because It is confusing. It is best to first explain the extraction process in full, and then continue with an explanation of purification. With a more logical and systematic sequence, readers can more easily understand the flow and stages.

**Response:** We appreciate the reviewer's insightful comment regarding the organization of the Introduction. We have carefully revised the section to address this concern. The text now presents a clear and logical progression, beginning with an overview of mangosteens and their bioactive compounds, followed by a comprehensive description of the extraction process. The purification methods are subsequently detailed. We believe this revised structure enhances the clarity and readability of the manuscript.

**Original:** Introduction

Mangosteen (Garcinia mangostana L.) is a significant commercial fruit in Thailand and various Southeast Asian countries, often referred to as the "queen of fruits" due to the crown-like appearance of its flowers. Notably, India leads the global production of mangosteens, while Thailand ranks second in Southeast Asia after Indonesia. The fruit's composition comprises 83% pericarps, 15% pulp, and 2% seeds by weight [1]. The pericarps, often considered byproducts, possess 82.50% carbohydrates, 6.45% fat, 3.02% protein, 2.17% ash, and 2.17% free sugars. [2]

Although the pericarp of fresh mangosteen initially holds a modest market value of 0.2 USD per kg, its value significantly increases when functional chemicals, particularly xanthones, are extracted from it. Studies suggest that from 1 kg of fresh mangosteen, approximately 2 g of pure xanthones can be obtained, which are valued at 15 times more than the pericarp itself [3]. Consequently, the extraction and purification procedures for xanthones are necessary for various industries, presenting a marketable product with substantial health advantages.

Mangosteen pericarps are rich in health-promoting phenolic compounds, including tannins, flavonoids, anthocyanins, xanthones, and benzophenones [4]. Xanthones, a subgroup of phenolics, are particularly abundant and are known for their diverse health benefits such as antitumor, anticancer, antibacterial, and antioxidant properties [5]. Approximately 200 varieties of xanthones exist in nature, with about 40 types found in mangosteen, belonging to families such as Bonnetiaceae, Clusiaceae, and Podostemaceae [6].

Extracts from mangosteen pericarp and xanthones find extensive applications across the medical, food supplement, oral care, and cosmetic industries. In medicine, pericarp extracts form the basis for various oral health products targeting fungal itch and ulcers. Within the food supplement sector, mangosteen pericarps are utilized in dietary supplements, including antioxidants and anti-aging products, to enhance immunity. The oral care industry employs pericarp extract in mouthwash, toothpaste, dental floss, and breath spray formulations. In the food industry, it contributes to antioxidant snacks, energy-boosting foods, snack bars, rice powder, and healthy beverages [7]. Furthermore, in cosmetics, pure xanthones derived from mangosteen pericarp are incorporated into skincare products designed to address acne and aging, promoting skin health and radiance [8].

Xanthones, polyphenol compounds abundant in mangosteen pericarp, include more than 68 varieties, with Alpha-mangostin (⍺-M) being the predominant substance (69.01%), followed by Gamma-mangostin (γ-M) (17.86%) and minor compounds. Figure 1 illustrates the chemical structures of xanthones and ⍺-M.

Xanthones can be extracted from either fresh or dried mangosteen pericarp. However, due to issues related to high moisture content and the risk of microbial contamination, dried pericarps are commonly preferred for extraction purposes [9]. Increased antioxidant capacity has been attributed to ⍺-M, a hydrophobic polyphenol compound [10, 11]. Therefore, purification is crucial to ensure the quality of target bioactive compounds, preventing undesirable side effects. The choice of solvent during extraction significantly influences compound purity, with various solvents of different polarities employed based on the characteristics of target compounds.

Solvent extraction, a technique to isolate desired compounds without dissolving others, is categorized into leaching (solid-liquid extraction) and liquid-liquid extraction. Factors affecting solid-liquid extraction include solvent and solid properties such as size and extraction time [12]. Although temperature affects solubility and diffusion rates, it does not influence viscosity; however, high temperatures may damage the target bioactive compounds. Solid-liquid extraction is commonly employed for extracting active substances from mangosteen pericarp. Various methods exist for extracting bioactive xanthones from mangosteen pericarp. Conventional extraction techniques such as maceration [13] and Soxhlet extraction [14] require substantial solvent volumes and prolonged extraction durations. Due to these drawbacks, several contemporary extraction methods have been explored to retrieve phenolic and bioactive compounds. For instance, microwave extraction with ethyl acetate and ultrasonic extraction with ethanol have shown promising results in extracting xanthones, notably ⍺-M. For instance, Ghasemzadeh et al. employed microwave extraction with 72.40% (v/v) ethyl acetate, yielding approximately 120.68 mg of xanthones/g of mangosteen pericarp (dry matter) [15]. Ultrasonic extraction with 95% (v/v) ethanol at 30 kHz and room temperature resulted in the extraction of α-mangostin and 8-desoxygartanin with a yield of 47.82 ± 3.76 mg/g and 1.43 ± 0.30 mg/g (dry basis), respectively [16]. Supercritical water extraction was explored within the temperature range of 120–180 °C and the pressure range of 1 to 5 MPa. The optimal xanthone yield reached 34 mg/g of the sample under conditions of 180 °C and 3 MPa, with a reaction time of 150 min [17]. Although advanced techniques such as supercritical carbon dioxide (SC–CO_2_) [18, 19], ultrasonic bath [12], and microwave-assisted extraction (MAE) [20] show significant α-mangostin extraction efficiency, they are costly and necessitate specialized equipment.

The quality of the targeted active compounds generated relies on employing an effective extraction method. The presence of a clearly defined chromatographic peak corresponding to the targeted chemical signifies the purity of the extracted substance. Ensuring the purity of a molecule derived from a plant extract is crucial for preventing undesirable side effects in pharmaceutical or medical uses, as variations in the compound's chemical properties can lead to adverse outcomes. Purification plays a critical role in the extraction process to yield a pure compound. To analyze the efficiency of the extraction procedure, a purified product can undergo further scrutiny using analytical techniques such as GC-MS [21], HPLC SFC [22], LC-MS [23] [24] [25], and UPLCMS [26].

In addition to securing a pure extract, the choice of an appropriate solvent during the extraction process is imperative for producing a high-purity compound as intended [27]. The selection of the solvent is also influenced by the characteristics of the targeted bioactive compound. Various solvents with different polarities are extensively employed to separate the target compound from a mixture based on their respective polar characteristics. Antioxidant components such as flavonoids, lignans, mitragynine, and xanthone from ethnobotanicals were extracted using solvents with varying polarity, according to prior work [28]. Similar methods using various polar solvents were used in another study to extract the main polyphenols from "misai kucing" (Orthosiphon stamineus), which showed a notable ability to scavenge free radicals [29].

For multi-stage extraction known as sequential extraction, different solvents with varying polarities are used to isolate the target compound from different substrates. The efficiency of this method is based on the polar attributes of compounds present in the raw material. Non-polar compounds such as fat, wax, pigments, fatty acids, alkane, sterols, and chlorophyll are initially extracted using a non-polar solvent (such as hexane or petroleum ether). To further solubilize the intermediate polar molecules, such as flavonoids and lipophilic chemicals, the intermediate polar solvents (such as dichloromethane and chloroform) are used. Eventually, alkaloids, tannins, and other hydrophilic chemicals are among the high-polar compounds that are usually extracted using high-polar solvents such as methanol and ethanol [30]. This method is potentially effective for the isolation of ⍺-M from mangosteen pericarps.

The efficient isolation of α-M from pericarp typically employs a three-stage sequential solvent approach, involving water, hexane, and acetonitrile. This methodology streamlines the extraction process, yielding α-M with elevated levels of purity and quantity, all without necessitating the utilization of sophisticated apparatus within the experimental framework. This investigation endeavors to formulate a direct and economical procedure for procuring α-M of superior quality, thereby obviating the requirement for specialized instrumentation or intricate methodologies commonly associated with traditional solvent extraction techniques. The arrangement of solvents in the extraction sequence was determined based on the solvent distribution factor. Subsequently, assessments were conducted to ascertain the yield, purity, and recovery of α-M. Finally, a precipitation technique was employed to generate solid α-M powder.

**Revised:** Introduction

Mangosteen (Garcinia mangostana L.), often hailed as the "queen of fruits" due to its distinctive floral appearance, is a commercially significant crop in Thailand and Southeast Asia. While India leads global production, Thailand is a major producer in the region [1]. The fruit comprises 83% pericarps, 15% pulp, and 2% seeds [2]. Although the pericarps have limited initial market value, they possess substantial potential due to their rich content of bioactive compounds, particularly xanthones [3]. These compounds exhibit a wide range of health benefits, including antioxidant and anticancer properties, driving demand across medical, food, cosmetic, and oral care industries [4, 5].

Mangosteen pericarps contain over 68 types of xanthones, with 69.01% of alpha-mangostin (⍺-M) and 17.86% of gamma-mangostin (γ-M) being the predominant compounds [6]. Alpha-mangostin, a hydrophobic polyphenol, is primarily responsible for the fruit's antioxidant capacity, highlighting the importance of purification processes to isolate this valuable compound [7]. Bioactive compounds can be extracted from mangosteen pericarp through solid-liquid extraction. While both fresh and dried pericarps are viable starting materials, the latter is generally preferred to minimize microbial contamination [8]. Factors affecting solid-liquid extraction include solvent and solid properties as well as the operating conditions such as extraction time, and temperature [9]. High temperatures can degrade bioactive compounds despite influencing solubility and diffusion rates. Various methods exist to extract bioactive xanthones from mangosteen pericarp. Conventional methods such as solvent extraction [10], maceration [11, 12, 13] and Soxhlet extraction [14] require substantial solvent volumes and prolonged durations. Due to these drawbacks, contemporary methods have been explored to retrieve phenolic and bioactive compounds. Microwave extraction with ethyl acetate and ultrasonic extraction with ethanol have shown promising results in extracting xanthones, notably ⍺-M. The extraction yield of ⍺-M and γ-M using methylene chloride as solvent was reported as 33.24 mg/g dried mangosteen (DM) and 8.61 mg/g of DM, respectively [6]. Ghasemzadeh et al. used microwave extraction with 72.40% (v/v) ethyl acetate, yielding 120.68 mg of xanthones/g of mangosteen pericarp (dry matter) [15]. Ultrasonic extraction with 95% (v/v) ethanol at 30 kHz and room temperature extracted α-mangostin and 8-desoxygartanin with yields of 47.82 ± 3.76 mg/g and 1.43 ± 0.30 mg/g (dry basis), respectively [16]. Supercritical water extraction was tested at 120–180 °C and 1 to 5 MPa. The optimal xanthone yield was 34 mg/g of the sample at 180 °C and 3 MPa, with a reaction time of 150 min [17]. Advanced techniques like supercritical carbon dioxide (SC–CO_2_) [18, 19], ultrasonic bath [9], and microwave-assisted extraction (MAE) [15] show significant α-mangostin extraction efficiency but are costly and require specialized equipment.

The quality of extracted bioactive compounds depends on an effective extraction method, with a clear chromatographic peak indicating purity. Ensuring purity is crucial to prevent side effects in pharmaceutical or medical applications, as chemical variations can lead to adverse outcomes. Purification plays a critical role in the extraction process to yield a pure compound. To analyze the efficiency of the extraction procedure, a purified product can undergo further scrutiny using analytical techniques such as Gas Chromatography/Mass Spectrometry (GC-MS) [20], Supercritical fluid chromatography (HPLC-SFC) [21], Liquid Chromatography - Mass Spectrometry (LC-MS) [22] [23] [24], and Ultra-performance liquid chromatography–mass spectrometry (UPLC-MS) [25].

For multi-stage extraction known as sequential extraction, different solvents with varying polarities are used to isolate the target compound from different substrates [26]. The efficiency of this method is based on the polar attributes of compounds present in the raw material. Non-polar compounds such as fat, wax, pigments, fatty acids, alkane, sterols, and chlorophyll are initially extracted using a non-polar solvent (such as hexane or petroleum ether) [27]. To further solubilize the intermediate polar molecules, such as flavonoids and lipophilic chemicals, the intermediate polar solvents (such as dichloromethane and chloroform) are used [28]. Eventually, alkaloids, tannins, and other hydrophilic chemicals are among the high-polar compounds that are usually extracted using high-polar solvents such as methanol and ethanol [29]. This method is potentially effective for the isolation of ⍺-M from mangosteen pericarps.

The efficient isolation of α-M from pericarp typically employs a three-stage sequential solvent approach, involving water, hexane, and acetonitrile. This methodology streamlines the extraction process, yielding α-M with elevated levels of purity and quantity, all without necessitating the utilization of sophisticated apparatus within the experimental framework. This investigation endeavors to formulate a direct and economical procedure for procuring α-M of superior quality, thereby obviating the requirement for specialized instrumentation or intricate methodologies commonly associated with traditional solvent extraction techniques. The arrangement of solvents in the extraction sequence was determined based on the solvent distribution factor. Subsequently, assessments were conducted to ascertain the yield, purity, and recovery of α-M. Finally, a precipitation technique was employed to generate solid α-M powder.

2. Materials & methods

- For the factors you mentioned regarding the content of the alpha mangostin compound in the pericarp, what function does this information have in your research? Do you also perform mangostin extraction on the pericarp, which is influenced by these factors? If yes, it also includes data regarding treatment. If there is any from other research, include data from that research

**Response:** We appreciate the reviewer’s inquiry regarding the relevance of factors influencing α-mangostin content in the pericarp to our study. Indeed, this information provides essential context for our research on α-mangostin extraction. As outlined in Sections 2.3, 2.4, and 2.5, our methodology focuses on solvent extraction of α-mangostin from the pericarp. The corresponding results are presented in Sections 3.1, 3.2, and 3.3. Furthermore, a comparative analysis of our extraction performance with existing literature is provided in Section 3.5. In the Introduction, the sentence “Factors affecting solid-liquid extraction include solvent and solid properties as well as the operating conditions such as extraction time, and temperature [9].” Provides a foundational overview of solid-liquid extraction, which are relevant to the present study.

- The formula used in this manuscript was inconsistent. If you want to use a list of information, you should place it below the formula. However, if you want to use paragraphs, use them consistently to explain the formula. Consistency in writing format will help readers to understand formulas and information more easily and clearly.

**Response:** We appreciate the reviewer’s insightful comment regarding the inconsistency in formula presentation. We have carefully reviewed the manuscript and standardized the format for explaining formulas. Specifically, we have adopted a consistent approach of using lists to present information related to equations (2) and (3). We believe these modifications enhance the clarity and readability of the mathematical content.

**Original:** Equation 2

$$m= \frac{y}{x}$$

(2)

where, m is the solvent distribution factor, $y$ is the mass fraction of ⍺-M in the solution and $x$ is the mass fraction of ⍺-M in the solute.

**Revised:** Equation 2

$$m= \frac{y}{x}$$

(2)

m = solvent distribution factor,

$y$ = mass fraction of ⍺-M in the solution,

$x$ = mass fraction of ⍺-M in the powder.

**Original:** Equation 3

| Y = $\frac{E y}{S_{0}}$ | (3) |
| --- | --- |

where Y is the yield, E is the volume of the extract solution, and y is a mass fraction of ⍺-M in solution and $S_{0}$ is the mass of the feed mangosteen powder.

**Revised:** Equation 3

| Y = $\frac{E y}{S_{0}}$ | (3) |
| --- | --- |

Y = the extraction yield of ⍺-M,

E = volume of the extract solution,

y = mass fraction of ⍺-M in solution,

$S_{0}$ = mass of the mangosteen powder.

3. Results and Discussion

- You stated that the mangostin taken was from the Bangkok market, but why do you mention a different source here? Please explain

**Response:** We appreciate you bringing this inconsistency to our attention. The mangosteen samples utilized in this study were indeed procured from Simumuang Market in Pathum Thani province, Thailand. To enhance clarity, we have removed the erroneous sentences in section 4.1.

**Original:** 4.1. Determination of mass fraction of ⍺-M inside the feed mangosteen powder

Due to the variation of ⍺-M content in the mangosteen pericarp obtained from different sources, it was necessary to determine the mass fraction of ⍺-M in our mangosteen pericarp powder. This was studied via crosscurrent extraction. The result was used to assess the %recovery of ⍺-M….

**Revised:** 3.1. Determination of mass fraction of ⍺-M inside the feed mangosteen powder

The α-M content was studied via crosscurrent extraction. The result was used to assess the %recovery of ⍺-M….

- The length of an abbreviation must be stated at the beginning of the abbreviation so that readers are not confused

**Response:** We thank the reviewer for their valuable comment regarding the clarity of abbreviations. We have carefully addressed this point by explicitly stating the full form of each abbreviation upon its first occurrence in the text. We believe these modifications enhance the readability and comprehension of the manuscript.

**Original:** Abstract

*Keywords:* alpha-mangostin (⍺-M), HPLC, sequential extraction, purity, yield

**Revised:** Abstract

Keywords: alpha-mangostin (⍺-M), High-Performance Liquid Chromatography (HPLC), sequential extraction, purity, yield

**Original:** Introduction

To analyze the efficiency of the extraction procedure, a purified product can undergo further scrutiny using analytical techniques such as GC-MS [21], HPLC SFC [22], LC-MS [23] [24] [25], and UPLCMS [26].

**Revised:** Introduction

To analyze the efficiency of the extraction procedure, a purified product can undergo further scrutiny using analytical techniques such as Gas Chromatography/Mass Spectrometry (GC-MS) [20], Supercritical fluid chromatography (HPLC-SFC) [21], Liquid Chromatography - Mass Spectrometry (LC-MS) [22] [23] [24], and Ultra-performance liquid chromatography–mass spectrometry (UPLC-MS) [25].

**Original:** 2.8 Precipitation of crude extract

The final solution extract from the sequential solvent was mixed with the DI water to induce precipitation.

**Revised:** 2.8 Precipitation of crude extract

The final solution extract from the sequential solvent was mixed with the deionized (DI) water to induce precipitation

- I see that you use reference journals 13 and 33 as the sources of the large number of alpha mangostin compounds that were successfully extracted. These two sources are research conducted in 2010, and there are no years. Therefore, research sources that go back at least 5 years are needed so that the novelty of the research is clearer

**Response:** We thank the reviewer for the insightful comment regarding the age of references supporting the extraction of alpha-mangostin compounds. We acknowledge that updating the literature is crucial to establish the novelty of our research. As suggested, we have carefully reviewed the referenced works. While reference 13 primarily focuses on the maceration process, it has been retained due to its relevance to the overall context. To address the reviewer’s concern about the age of references, we have incorporated a more recent study (reference 34) published in 2021, which provides additional support for the extraction methodology. Furthermore, reference 33 has been corrected and supplemented with a contemporary study (reference 15) to strengthen the foundation of our work.

**Original:** 3.5 Comparison of extraction performance

Based on the literature review, the performance of conventional extraction of mangosteen pericarp was compared with our sequential extraction as presented in Table 3. Maceration, a widely used approach, yields 1.19 mg/g of xanthones from mangosteen pericarp when macerated with 95% ethanol for seven days [13]. Percolation, a continuous method, yields 12.71% w/w α-M when using a percolator with 95% ethanol. Soxhlet extraction, a traditional technique, suffers from drawbacks such as large solvent volumes and extended extraction periods [35]. Soxhlet extraction yielded a crude extract comprising 26.60% of the dry weight, with α-mangostin constituting 13.51% (w/w of the crude extract), achieved using a concentration of 50% ethanol [35]. Conventional methods such as maceration, soxhlet extraction, and heat reflux are time-consuming while offering low product recovery. Therefore, our three-stage or five-stage percolation technique can be an efficient alternative to recover α-M from mangosteen pericarp. The process is simple, and processing can be completed in a short time. Note that the solvent used in the extraction process can be recovered. Hence, the cost of solvent can be greatly reduced. Note that, unlike conventional extraction processes, our sequential extraction also yielded 46.75 mg/g α-M from mangosteen pericarp with a purity of α-M of 67.9%. Achieving high purity ensures that the extracted compound maintains its biological activity, allowing for accurate evaluation of its potential health-promoting properties.

**Table 3.** Extraction performance of different techniques

| Extraction Method | Conditions | Active Compounds Yield |
| --- | --- | --- |
| Maceration  [13] | macerated with 95% ethanol for seven days | yields 1.19 mg/g of xanthones from mangosteen pericarp |
| Percolation  [33] | using a percolator with 95% ethanol | 12.71% w/w α-M |
| Soxhlet extraction  [33] | using a concentration of 50% ethanol | crude extract (26.60% dry weight),  α-mangostin (13.51%, w/w of crude extract) |
| Proposed Method | Sequential Solvent Extraction (Water, Hexane, Acetonitrile) | yields 46.75 mg/g α-M with 67.9% purity from mangosteen pericarp |

[13] Pojanaukij N, Kajorncheappunngam S, Comparison of antimicrobial activity of mangosteen crude, turmeric and Gotu Kola extract. NUJST 2010, 18, 1–9.

[33] M. C. Bellissent-Funel, M. G. C. Longinotti, C. A. Angell, and C. Alba-Simionesco, Structure and dynamics of water-acetonitrile mixtures by computer simulations.

**Revised:** 3.5 Comparison of extraction performance

Based on the literature review, the performance of conventional extraction of mangosteen pericarp was compared with our sequential extraction as presented in Table 3. Maceration, a widely used approach, yields 1.19 mg/g of xanthones from mangosteen pericarp when macerated with 95% ethanol for seven days [13]. Tran et al. (2021) reported a xanthone yield of 5.626 mg/g from mangosteen pericarp after 96 h of ethyl acetate maceration of peel powder [34]. It is worth noting that ethyl acetate is less polar than ethanol due to its ester functional group. Microwave-assisted extraction using ethyl acetate as a green solvent achieved the highest α-mangostin (α-M) yield at the optimal conditions of 189.20 W power, 72.40% (v/v) ethyl acetate, and the extraction time of 3.16 min. The α-M content in the extract reached 121.01 mg/g dry matter (DM) [15]. However, the sophisticated equipment requirements, energy consumption, and maintenance associated with this technique represent significant drawbacks compared to other methods. Percolation, a continuous method, yields 12.71% w/w α-M when using a percolator with 95% ethanol. Soxhlet extraction, a traditional technique, suffers from drawbacks such as large solvent volumes and extended extraction periods [35]. Soxhlet extraction yielded a crude extract comprising 26.60% of the dry weight, with α-mangostin constituting 13.51% (w/w of the crude extract), achieved using a concentration of 50% ethanol [35]. Conventional methods such as maceration, soxhlet extraction, and heat reflux are time-consuming while offering low product recovery. Therefore, our three-stage or five-stage percolation technique can be an efficient alternative to recover α-M from mangosteen pericarp. The process is simple, and processing can be completed in a short time. Note that the solvent used in the extraction process can be recovered. Hence, the cost of solvent can be greatly reduced. Note that, unlike conventional extraction processes, our sequential extraction also yielded 46.75 mg/g α-M from mangosteen pericarp with a purity of α-M of 67.9%. Achieving high purity ensures that the extracted compound maintains its biological activity, allowing for accurate evaluation of its potential health-promoting properties.

**Table 3.** Extraction performance of different techniques

| Extraction Method | Conditions | Active Compounds Yield |
| --- | --- | --- |
| Maceration  [13] | macerated with 95% ethanol for 7 days | yields 1.19 mg/g of xanthones from mangosteen pericarp |
| Maceration  [34] | 500 g of peel powder macerated with 1L of ethyl acetate for 96 h | yields 5.626 mg/g of xanthones from mangosteen pericarp |
| Microwave [15] | Ethyl acetate as a green solvent followed by dichloromethane, ethanol, and water exhibited the highest α-M at 3.16 min, 189.20 W, and 72.40% (v/v). | α-M concentration in mangosteen pericarp of 121.01 mg/g dry matter (DM) |
| Percolation  [35] | using a percolator with 95% ethanol | 12.71% w/w α-M |
| Soxhlet extraction  [35] | using a concentration of 50% ethanol | crude extract (26.60% dry weight),  α-mangostin (13.51%, w/w of crude extract) |
| Proposed Method | Sequential Solvent Extraction (Water, Hexane, Acetonitrile) | yields 46.75 mg/g α-M with 67.9% purity from mangosteen pericarp |

[13] Pojanaukij N., and Kajorncheappunngam S., Comparison of antimicrobial activity of mangosteen crude, turmeric and Gotu Kola extract. NUJST, 2010. 18: p. 1–9.

[15] Ghasemzadeh A., Jaafar H.Z.E., Baghdadi A., and Tayebi-meigooni A., Alpha-mangostin-rich extracts from mangosteen pericarp: Optimization of green extraction protocol and evaluation of biological activity. Molecules, 2018. 23: p. 1852.

[34] Tran V.A., Vo T.T., Nguyen M.N.T., Dat N.D., Van-Dat N., Quang T.Q., Hieu V.Q., Thuan L.V., Danh T.T., Novel α-Mangostin Derivatives from Mangosteen (Garcinia mangostana L.) Peel Extract with Antioxidant and Anticancer Potential. J. Chem., 2021. 9985604: p. 12.

[35] Pothitirat W., Chomnawang M.T., Supabphol R., and Gritsanapan W., Free radical scavenging and anti-acne activities of mangosteen fruit rind extracts prepared by different extraction methods. Pharm. Biol., 2010. 48: p. 182–186.

4. Conclusion

- The conclusions presented by the researcher need to be shortened again

**Response:** Thank you for your suggestion. The conclusion has been revised accordingly.

**Original:** Conclusion

Alpha-mangostin was the bioactive compound extracted from mangosteen pericarp. Different solvents including water, hexane, and acetonitrile were used for extraction of α-M. According to the solvent distribution factor, a 3-stage sequential extraction was developed to effectively remove impurity and achieve the yield of 28.94 mg/g. Two additional stages using acetonitrile as solvent could improve the yield and recovery further to 46.75 mg/g and 73.05%, respectively. The increase in purity to 67.9% underscores the effectiveness of this approach in improving both recovery rates and purity levels. These collective findings highlight the potency of the devised sequential solvent extraction method in not only enhancing recovery rates but also improving the purity of extracted compounds, thus ensuring the preservation of their biological activity. Moreover, the precipitation method offers a promising alternative to traditional extraction techniques, offering a simple and efficient recovery of extracted product. This study provides valuable insights that can be used as a basis for further development of large-scale process for the extraction of α-M from mangosteen pericarps.

**Revised:** Conclusion

Alpha-mangostin, a valuable bioactive compound, was successfully extracted from mangosteen pericarp through an optimized sequential solvent extraction process. By systematically employing water, hexane, and acetonitrile, we achieved a significant enhancement in both yield (46.75 mg/g) and purity (67.9%) of alpha-mangostin compared to traditional methods. The incorporation of additional acetonitrile extraction stages proved instrumental in improving recovery rates and eliminating impurities. The precipitation method demonstrated efficacy in recovering the extracted product. This study provides a robust foundation for the development of a scalable process for the production of high-purity alpha-mangostin from mangosteen pericarp.

5. Bibliography

- 94% of the references used in this article came from articles published more than five years ago. This value makes the novelty of this research questionable. Therefore, researchers must look for other reference sources to replace the references that have been used

- Some references do not have a year, such as reference numbers 33 and 34. This should be corrected again

**Response**: We appreciate the reviewer’s valuable feedback regarding the age of our cited references. To enhance the manuscript’s novelty, we have conducted a thorough review of the reference list. A significant number of older references (specifically, references 3, 4, 6, 7, 9, 10, 11, 19, 21, 23, 24, 25, 26, 33, and 34) have been removed. Additionally, we have updated several references to incorporate more recent findings. All references now include accurate publication years. We are confident that the revised reference list effectively positions our research within the contemporary scientific landscape.

**Original:** Reference

[3] Sukatta U, Xanthones from Mangosteen Peels in the Cosmetic Industry. Available online: https://www3.rdi.ku.ac.th/?p=62656

[4] Kuma D, Sekar S, Analysis of physio-chemical properties of mangosteen rind extract from industrial waste. IJTSRD 2018, 2, 1522–1526.

[6] Chudhangkura A, Xanthones in mangosteen. Food 2015, 45, 37–40

[7] Quality Plus Aesthetic International Co., Ltd. Purified Xanthone extract from Mangosteen Peel. Available online: https://www.qualityplus.co.th/quality-plus-deep-technology/deep-biotechnology/purify-xanthone/ (accessed on 30 January 2024).

[9] Neelapong W, Phonyotin B, Sittikijyothin W, Extraction of active compounds from Thai herbs: Powder and extract. KMUTNB. 2019, 29, 157–166.

[10] Jung H-A, Su B-N, Keller WJ, Mehta RG, Kinghorn AD, Antioxidant xanthones from the pericarp of Garcinia mangostana (Mangosteen). J. Agric. Food Chem. 54 (6), 2077–2082, 2006.

[11] Suvarnakuta P, Chaweerungrat C, Devahastin S, Effects of drying methods on assay and antioxidant activity of xanthones in mangosteen rind. Food Chem. 125 (1), 240–247, 2011.

[19] Zarena A.S, Manohar B, Udaya Sankar K, Optimization of supercritical carbon dioxide extraction of xanthones from mangosteen pericarp by response surface methodology. Food Bioprocess Technol. 5 (4), 1181–1188, 2012.

[21] A. A. Philipp, M. R. Meyer; D. K. Wissenbach, A. A. Weber, S. W. Zoerntlein, P. G. M. Zweipfenning, H. H. Maurer, Monitoring of kratom or krypton intake in urine using GC-MS in clinical and forensic toxicology, Anal. Bioanal. Chem. vol. 400, no. 1, pp. 127-135, 2011.

[23] K. B. Chan, C. Pakiam, R. A. B. Rahim, Psycoactive plant abuse: The identification of mitragynine in ketum and in ketum preparations, Narcotics. vol. 57, no. 2, pp. 249-256, 2005.

[24] A. A. Philipp, D. K. Wissenbach, A. Weber, J. Zapp, S. W. Zoerntlein, K. Kanogsunthornrat, H. H. Maurer, Use of liquid chromatography coupled to low- and high-resolution linear ion trap mass spectrometry for studying the metabolism of paynantheine, an alkaloid of the herbal drug kratom in rat and human urine, Anal. Bioanal. Chem. vol. 396, no. 7, pp. 2379-2391, 2010.

[25] S. Lu, B. N. Tran, J. L. Nelsen, K. M. Aldous, Quantitative analysis of mitragynine in human urine by high performance liquid chromatography-tandem mass spectrometry, J Chromatogr. B: Anal. Technol. Biomed. Life Sci. vol. 877, no. 24, pp. 2499-2505, 2009.

[26] P. K. Vuppala, S. Jamalapuram, E. B. Furr, C. R. McCurdy, Development and validation of a UPLC‐MS/MS method for the determination of 7‐hydroxymitragynine, a μ‐opioid agonist, in rat plasma and its application to a pharmacokinetic study. Biomed. Chromatogr. vol. 27, no. 12, pp. 1726-1732, 2013.

[33] M. C. Bellissent-Funel, M. G. C. Longinotti, C. A. Angell, and C. Alba-Simionesco, Structure and dynamics of water-acetonitrile mixtures by computer simulations.

[34] Skoog D.A, Holler F.J, Crouch S.R, Holler F, Principles of instrumental analysis, Thomson Brooks/Cole.

**Revised:** Reference

[1] Adriani L., Widjastuti T., Nurdianti R.R., and Wiradimadja R., Effects of mangosteen peel extract (Garcinia mangostana L.) on blood lipid of ntiox chicken growth phase. In Proceedings of the 3rd International Conference of Integrated Intellectual Community (ICONIC), Hannover, Germany, 27–29 April 2018; pp. 1–5.

## [2] Charoenphun N., Setarnawat S., and Sai-Ut S., Chemical composition and trends in utilization of by-products and wastes from 4 types of tropical fruit processing. TJST, 2020. 28: p. 113–128.

## [3] Mello R.F.A., Pinheiro W.B.S., Benjamim J.K.F., Siqueira F.C., Chiste R.C., and Santos A.S., A fast and efficient preparative method for separation and purification of main bioactive xanthones from the waste of Garcinia mangostana L. by high-speed countercurrent chromatography. Arab. J. Chem., 2021. 14: p. 103252.

## [4] Widowati W., Ginting C.N., Lister N.E., Gisang E., Amalia A., Wibowo S.H.B., Kusuma H.S.W., and Rizal, Anti-aging effects of mangosteen peel extract and its phytochemical compounds: Antioxidant activity, enzyme inhibition and molecular docking simulation. Trop. Life Sci. Res., 2020. 31: p. 127–144.

## [5] Chaiwong N., Phimolsiripol Y., Leelapornpisid P., Ruksiriwanich W., Jantanasakulwong K., Rachtanapun P., Seesuriyachan P., Leksawasdi N., Simirgiotis M.J., Barba F.J., et al., Synergistics of carboxymethyl chitosan and mangosteen extract as enhancing moisturizing, antioxidant, antibacterial, and deodorizing properties in emulsion cream. Polymers, 2022. 14: p. 178.

[6] Wittenauer J., Falk S., Schweiggert-Weisz U., and Carle R., Characterisation and quantification of xanthones from the aril and pericarp of mangosteens (Garcinia mangostana L.) and a mangosteen containing functional beverage by HPLC-DAD-MS. Food Chem., 2012. 134(1): p. 445–452.

[7] Janardhanan S., Mahendra J., Girija A.S., Mahendra L., and Priyadharsini V., Antimicrobial effects of Garcinia mangostana on cariogenic microorganisms. J. Clin. Diagn. Res., 2017. 11: p. ZC19–ZC22.

[8] Asasutjarit R., Meesomboon T., and Adulheem P., Physicochemical properties of alpha-mangostin loaded nanomeulsions prepared by ultrasonication technique. Heliyon, 2019. 5(9): art. no. e02465.

[9] Megawati, Megawati, Ginting R., Kusumaningtyas R., and Sediawan W., Mangosteen Peel Antioxidant Extraction and Its Use to Improve the Stability of Biodiesel B20 Oxidation. 2020.

[10] Bundeesomchok K., Filly A., Rakotomanomana N., Panichayupakaranant P., and Chemat F., Extraction of ±-mangostin from Garcinia mangostana L. using alternative solvents: computational predictive and experimental studies. LWT– Food Sci. Technol., 2016. 65: p. 297–303.

[11] Tiang N., Ahad M.A., Murugaiyah V., and Hassan Z., Xanthone-enriched fraction of Garcinia mangostana and α-mangostin improve the spatial learning and memory of chronic cerebral hypoperfusion rats. J. Pharm. Pharmacol., 2020. 72(11): p. 1629–1644.

[12] Muchtaridi M., Prasetio M., Mekar Saptarini N., and Amelia Saputri F., High performance liquid chromatography for α-mangostin analysis in mangosteen pericarp extract for routine analysis with photodiode array detector. Rasayan J. Chem., 2018. 11: p. 973–978.

[13] Pojanaukij N., and Kajorncheappunngam S., Comparison of antimicrobial activity of mangosteen crude, turmeric and Gotu Kola extract. NUJST, 2010. 18: p. 1–9.

[14] Yoswathana N., and Eshtiaghi M.N., Optimization of subcritical ethanol extraction for xanthone from mangosteen pericarp. Int. J. Chem. Eng. Appl., 2015. 6: p. 115–119.

[15] Ghasemzadeh A., Jaafar H.Z.E., Baghdadi A., and Tayebi-meigooni A., Alpha-mangostin-rich extracts from mangosteen pericarp: Optimization of green extraction protocol and evaluation of biological activity. Molecules, 2018. 23: p. 1852.

[16] Suvarnakuta P., Chaweerungrat C., and Devahastin S., Effects of drying methods on assay and antioxidant activity of xanthones in mangosteen rind. Food Chem., 2011. 125: p. 240–247.

[17] Machmudah S., Shiddiqi Q.Y.A., Kharisma A.D., Widiyastuti, Wahyudiono, Kanda H., Winardi S., and Goto M., Subcritical water extraction of xanthone from mangosteen (Garcinia mangostana Linn) pericarp. J. Adv. Chem. Eng., 2015. 5: p. 117.

[18] Chhouk K., Quitain A.T., Gaspillo P.D., Maridable J.B., Sasaki M., Shimoyama Y., and Goto M., Supercritical carbon dioxide-mediated hydrothermal extraction of bioactive compounds from Garcinia Mangostana pericarp. J. Supercrit. Fluids, 2016. 110: p. 167–175.

[19] Mishima K., Kawamura H., Ito S., Inoue Y., Hirota T., Kato T., Sharmin T., Kawakami R., Irie K., Mishima K., Harada T., Mustofa S., Hasanah F., Siregar Y.D.I., Putri L.S.E., and Salim A., Liquid carbon dioxide extraction of xanthones from the pericarps of Garcinia mangostana linn, Enhanced by Ultrasonic Irradiation. 2015. 22: p. 199.

[20] Mamat S.F., Azizan K.A., Baharum S.N., Noor N.M., and Aizat W.M., GC-MS and LC-MS analyses reveal the distribution of primary and secondary metabolites in mangosteen (Garcinia mangostana Linn.) fruit during ripening. Scientia Horticulturae, 2020. 262: art. no. 109004

[21] Wang M., Carrell E.J., Ali Z., Avula B., Avonto C., Parcher J.F., and Khan I.A., Comparison of three chromatographic techniques for the detection of mitragynine and other indole and oxindole alkaloids in Mitragyna speciosa (kratom) plants. J. Sep. Sci., 2014. 37(12): p. 1411-1418.

[22] Nawawi N.I.M., Ijod G., Abas F., Ramli N.S., Mohd Adzahan N., and Mohamad Azman E., Influence of Different Drying Methods on Anthocyanins Composition and Antioxidant Activities of Mangosteen (Garcinia mangostana L.) Pericarps and LC-MS Analysis of the Active Extract. Foods, 2023. 12: p. 2351.

[23] Netravati S., Gomez S., Pathrose B., Joseph M., Shynu M., and Kuruvila B., Comparison of extraction methods on anthocyanin pigment attributes from mangosteen (Garcinia mangostana L.) fruit rind as potential food colourant. Food Chem. Adv., 2024. 4: art. no. 100559.

[24] Liang X., Hu Y., et al., Identification and Pharmacokinetics of Quinone Reductase 2 Inhibitors after Oral Administration of Garcinia mangostana L. Extract in Rat by LC–MS/MS. J. Agric. Food Chem., 2020. 68(43): p. 11975-11986.

[25] Li R., Inbaraj B.S., and Chen B.H., Quantification of Xanthone and Anthocyanin in Mangosteen Peel by UPLC-MS/MS and Preparation of Nanoemulsions for Studying Their Inhibition Effects on Liver Cancer Cells. Int. J. Mol. Sci., 2023. 24(4): p. 3934.

[26] Takayama H., and Maruyama T., Simultaneous analysis of mitragynine, 7-hydroxymitragynine, and other alkaloids in the psychotropic plant “kratom” (Mitragyna speciosa) by LC-ESI-MS. Forensic Toxicol., 2009. 27: p. 67-74.

[27] Norazah M.A., Rahmani M., Khozirah S., Ismail H.B.M., Sukari M.A., and Ali A.M., Chemical constituents and antioxidant activity of Cinnamomum microphyllum. Pertanika J. Sci. Technol., 2010. 18(2): p. 263-270.

[28] Akowuah G.A., Ismail Z., Norhayati I., and Sadikun A., The effects of different extraction solvents of varying polarities on polyphenols of Orthosiphon stamineus and evaluation of the free radical-scavenging activity. Food Chem., 2005. 93(2): p. 311-317.

[29] Seidel V., Initial and Bulk Extraction of Natural Products Isolation. In: Sarker S.D., Nahar L., eds. Natural Products Isolation. Springer Science and Business Media, 2012. pp. 27-41.

[30] Romdhane M., and Gourdou C., Investigation in solid-liquid extraction: influence of ultrasound. Chem. Eng. J., 2002. 87(1): p. 11-19.

[31] Lin F., and Giusti M.M., Effects of solvent polarity and acidity on the extraction efficiency of isoflavones from soybeans (Glycine max). J. Agric. Food Chem., 2005. 53(10): p. 3795-3800.

[32] Zhao L., Ma K., and Yang Z., Changes of Water Hydrogen Bond Network with Different Externalities. Int. J. Mol. Sci., 2015. 16(4): p. 8454-8489.

[33] Skoog D.A., Holler F.J., and Crouch S.R., Principles of Instrumental Analysis. Sixth Edition. Brooks Cole, Belmont, 2007. p. 1039.

[34] Tran V.A., Vo T.T., Nguyen M.N.T., Dat N.D., Van-Dat N., Quang T.Q., Hieu V.Q., Thuan L.V., Danh T.T., Novel α-Mangostin Derivatives from Mangosteen (Garcinia mangostana L.) Peel Extract with Antioxidant and Anticancer Potential. J. Chem., 2021. 9985604: p. 12.

[35] Pothitirat W., Chomnawang M.T., Supabphol R., and Gritsanapan W., Free radical scavenging and anti-acne activities of mangosteen fruit rind extracts prepared by different extraction methods. Pharm. Biol., 2010. 48: p. 182–186.
